# Tea for histamine anti-allergy: component analysis of tea extracts and potential mechanism for treating histamine anti-allergy

**DOI:** 10.3389/fphar.2024.1296190

**Published:** 2024-05-30

**Authors:** Zeting Huang, Lanyue Zhang, Jie Xuan, Lu Yang, Tiantian Zhao, Weihua Peng

**Affiliations:** ^1^ Guangzhou Zhongzhuang Meiye Cosmetics Co Ltd., Guangzhou, China; ^2^ School of Biomedical and Pharmaceutical Sciences, Guangdong Provincial Key Laboratory of Plant Resources Biorefinery, Guangdong University of Technology, Guangzhou, China; ^3^ Sericulture and Agri-food Research Institute Guangdong Academy of Agricultural Sciences, Key Laboratory of Functional Foods, Ministry of Agriculture and Rural Affairs, Guangdong Key Laboratory of Agricultural Products Processing, Guangzhou, China; ^4^ Department of Food Science, Rutgers University, New Brunswick, NB, United States

**Keywords:** camellia, chemical compositions, histamine, anti-allergic dermatitis, anti-oxidation, anti-inflammation

## Abstract

In China, *Camellia* plants are widely used to reduce atopic dermatitis and inflammation-related diseases, but their protective mechanisms remain unclear. This study investigated the anti-allergic dermatitis, anti-oxidation and anti-inflammation effect and underlying mechanism of five *Camellia* species, including *Camellia ptilophylla Chang*, *Camellia assamica Chang* var. *Kucha Chang*, *Camellia parvisepala Chang*, *Camellia arborescens Chang*, and *C*. *assamica M. Chang*. A total of about 110 chemical compositions were detected from five *Camellia* teas extracts. The level of mast cell infiltration in the model mice skin was determined by HE (Hematoxylin and eosin) staining and toluidine blue staining, and the level of interleukin-1*β* (IL-1β) and nerve growth factor was detected by immunohistochemistry. The five Camellia tea leaf extracts have histamine-induced allergic dermatitis. Lipopolysaccharide (Lipopolysaccharide)-induced murine macrophage RAW264.7 inflammation model was found to secrete NF-κB factor, as shown by immunofluorescence, and reactive oxygen species secretion and related cytokine levels were detected. The results suggested that Camellia’s five tea extracts had the ability to resist cellular oxidative stress. In addition, the results of cell inflammatory cytokines including fibronectin (FN) and interleukin-6 (IL-6) suggested that the five tea extracts of *Camellia* had anti-inflammatory effects. Therefore, it is suggested that five *Camellia* teas may possess inhibitory properties against allergic reactions, oxidative stress, and inflammation, and may prove beneficial in the treatment of allergies.

## 1 Introduction

Skin is an important immunological organ and often experiences allergic and immunological responses. Skin hypersensitivity, also known as allergic reaction, refers to the specific immune response of the human immune system to specific allergens, such as tissue cell injury or physiological dysfunction (Park et al., 2018). Skin hypersensitivity is the most common type of hypersensitivity mediated by lgE (Feinberg, 2018), where mast cells act as the main response cells (Khan and Kemp, 2011; Hagemann et al., 2019). The mechanism is that antigens enter the body and bind to lgE attached to mast cells and basophils (Gilfillan and Tkaczyk, 2006; Wernersson and Pejler, 2014), inducing cells to be activated and release histamine, 5-hydroxytryptamine and other bioactive substances (Nitschke et al., 1996; Zeng et al., 2023). Histamine is the main chemical mediator of immune and inflammatory responses (Li H. et al., 2022; Ke et al., 2023). As a major factor in allergic diseases, histamine is present in cytoplasmic granules of human basophilic granulocytes and mast cells (Yasuda et al., 2019), and exists in extracellular regions of the epidermis (Paivandy and Pejler, 2021). When allergic reaction occurs in tissues, it will cause the rupture of basophilic granulocytes and mast cells to release histamine, and induce keratinocytes to secrete inflammatory cytokines IL-1β and NGF (Lipnik-Stangelj et al., 2013). In addition, it will also cause dilation of capillaries and venules and increase permeability of tube walls, smooth muscle spasm, and enhancement of glandular secretion activities, resulting in local skin tissue showing congestion, erythema, and swelling (Maria Arin et al., 2017; Gao et al., 2018; Yamamoto et al., 2022). Existing studies have shown that histamine secretion can stimulate the body to produce ROS and trigger oxidative stress in the body (Hassanen et al., 2023), and promote the release of inflammatory factors through the NF-*κ*B pathway (Park et al., 2014).

*Camellia ptilophylla Chang*, *Camellia assamica Chang* var. *Kucha Chang*, *Camellia parvisepala Chang*, *Camellia arborescens Chang*, and *C. assamica M. Chang* are all plants of the Theaceae family and *C*. genus. Tea contains more than 500 compounds, rich in tea polyphenols, alkaloids, tea polysaccharides, theanine, tea saponins and other biochemical components (Li S. et al., 2022; Huang et al., 2023; Lin et al., 2023; Tzen, 2023). There is evidence that it has anti-allergic, anti-inflammatory, antioxidant, and antibacterial properties. Tea extract has immunomodulatory activity (Matsuda et al., 2012; Matsuda et al., 2012; Yang et al., 2020; Wang et al., 2022), which can participate in inflammatory response by inhibiting NF-κB signaling pathway and inducing macrophages to produce various inflammatory cytokines (Moosa et al., 2018; Al-Khayri et al., 2022; Wang et al., 2023).

According to this study, five tea extracts were obtained: *C. ptilophylla Chang* (CPt), *C*. *assamica C.* var. *Kucha Chang* (CAk), *C*. *parvisepala Chang* (CPa), *C*. *arborescens Chang* (CAr), *C*. *assamica M. Chang* (CAm). The effective components of these extracts were identified by UPLC-Q-TOF, and the anti-inflammatory and anti-free radical activities of the extract were evaluated. Through histological analysis of the experimental mouse model, HE staining, staining the skin with toluidine blue, mast cells were detected in the tissues of experimental mice, and the level of proinflammatory factor was detected by immunohistochemistry. In the mouse macrophage RAW264.7 inflammation model induced by LPS, the secretion of NF-*κ*B factor was detected by immunofluorescence, and the secretion of ROS and the level of related cytokines were detected, results suggested that the five tea extracts of *Camellia* had the ability of resisting cellular oxidative stress. In addition, the results of cell inflammatory cytokines including FN and IL-6 detected by ELISA suggested that five *Camellia* tea extracts had anti-inflammatory effects. Overall, this study shows that five Camellia teas inhibit allergies, oxidative stress, and inflammation, so they may be candidates for treating allergies.

## 2 Materials and methods

### 2.1 Reagents and plant materials

H&E (Hematoxylin-Eosin), toluidine blue, and IHC (Immunocyto-Chemistry) kits were procured from Servicebio (Wuhan, China). Fibronectin (FN) ELISA kit, interleukin-6 (IL-6) ELISA kit, neutral red cell proliferation and cytotoxicity assay kit and nuclear factor kappa B (NF-*κ*B) assay kit were obtained from Beyotime (Shanghai, China). MTT kit and ROS kit were purchased from Solarbio (Beijing, China). Phosphate buffered saline (PBS) and culture medium were purchased from Gibco (Shanghai, China). Lipopolysaccharide (LPS) was obtained from Sigma-Aldrich (Shanghai, China). Automatic digital slide scanning system (model: Axio Scan. Z1) was from Zeiss (Carl Zeiss AG, Germany). The inverted fluorescence microscope (model: Olympus IX71) was from Olympus (Olympus Corporation, Japan). The CO_2_ cell culture incubator (Model: CellXpert C170i) was from Eppendorf (Eppendorf AG, Germany). Cell counter was from corning (Corning Incorporated, United States). *Camellia ptilophylla Chang*, *C. assamica C.* var. *Kucha Chang*, *C. parvisepala Chang*, *C. arborescens Chang*, and *C. assamica M. Chang* were collected from five local tea factories in southeastern China.

### 2.2 Extraction method of tea extract and drug preparation for treating mice

First, five kinds of *Camellias* were washed with deionized water, freeze-dried them, ground them, and passed through a 100 mesh sieve. 20 g of each of the five kinds of *Camellias*, 300 mL of 75% ethanol, extracted at 45°C for 30 min in the ultrasonic extraction equipment, centrifuged for 10 min, 300 mL of 75% ethanol was added to the filter residue, put into the ultrasonic extractor for another 30 min, centrifuged for 10 min, combined with the filtrate and concentrated under reduced pressure, 50 mL of extract was obtained, and it was refrigerated at 4°C for testing. Treatment of mice is 0.5% *Camellia* extract diluted with normal saline.

### 2.3 Chemical constituents of five tea extracts analyzed by UPLC-Q-TOF

In order to detect the main components of five species of *Camellia* plants and explore their active ingredient on treating histamine anti-allergy, their extracts were analyzed by UPLC-Q-TOF (Thermal, Ultimate 3000LC, and Q Exactive HF) instrument analysis platform and C18 column (Zorbax Eclipse C18 (1.8 μm*2.1*100 mm). The chromatographic separation conditions were as follows: column temperature is 30°C; Flow rate is 0.3 mL/min; mobile phase consisted of water +0.1% formic acid and pure acetonitrile. The sample size was 2 μL and the temperature of the automatic injector was 4°C. Positive mode: heater temperature 325°C; gas flow rates: sheath gas at 45 bar; auxiliary gas at 15 bar; purge gas at 1 bar; spray voltage: 3.5 KV; capillary temperature: 330°C; S-Lens RF Level: 55%. Negative mode: heater temperature 325°C; sheath gas flow rate is 45 bar; auxiliary gas flow rate is 15 arb; purge gas flow rate is one arb; electric spray voltage is 3.5 KV; capillary temperature is 330°C; S-Lens RF Level is 55%. Scanning mode: full primary scan (m/z 100–1,500) and secondary data-dependent mass spectrometry scan (dd-MS2, TopN = 10); Resolution: 70,000 (primary mass spectrometry) and 17,500 (secondary mass spectrometry). Collision mode: High energy collision dissociation (HCD).

### 2.4 Animals and treatment

To evaluate the effects of *Camellia* extract on mouse skin, preconfigured *Camellia* extract was applied. Fourty-two male KM mice (mean weight 21 g over 5 weeks) were obtained from Guangdong Medical Laboratory Animal Center (SCXK/2013–0,002). In addition, the University’s Animal Experiment Ethics Committee approved procedures for animal experimentation. One day before the experiment, the hair on the back of the mice was cut short, and the depilatory area was about 4 cm × 4 cm. The trial was divided into a 1-week pre-administration phase and an acute sensitization phase. During the early administration phase, the model group and blank control group received propylene glycol solution (200 μL/piece) on the back hair removal site, the positive control group was coated with 0.5% propylene glycol hyaluronate solution (200 μL/piece) on the back hair removal site, and the single and compound groups of 0.5% were coated with the corresponding drug (200 μL/piece) on the back hair removal site. All of the above groups were given over a 7-day period, once a day.

A mouse model of histamine-induced acute pruritus was established on the seventh day of the trial. All groups of mice were first administered, the concentration of the drug was 0.5% *Camellia* extract diluted with normal saline, and the positive group received diphenhydramine hydrochloride intraperitoneally (dissolved in normal saline solution, dose of 2 mg/mL, administered at a dose of 10 g). After administration for 30 min, 100 mL of normal saline was injected intraperitoneally in the blank group, 100 mL of histamine (dissolved in normal saline solution at 1 mg/mL concentration) was injected intraperitoneally in the other groups. Video recordings were then used to determine the number of scratches in each group. After that, the mice were killed by cervical dislocations. The skin tissues of the back were fixed and frozen in paraformaldehyde (two tubes each), and subsequent indicators were determined.

### 2.5 Hematoxylin and eosin staining (HE)

Sections of skin tissue were made of paraffin and stained with hematoxylin and eosin. It was then captured under a microscope and randomly selected areas were used to collect images for histological analysis.

### 2.6 Toluidine blue staining

In the process of dewaxing to water, paraffin sections were rinsed with water, submerged in a dye solution for 2–5 min, differentiated with 0.1% glacial acetic acid, and dried, and transparently sealed. Microscopic examination, image collection and analysis.

### 2.7 Immunohistochemistry analysis

Primary (Anti-NGF Rabbit pAb and Anti-IL-1*β* Rabbit pAb) and secondary antibodies (HRP-labeled goat-anti-rabbit IgG) were added to the immunohistochemical sections. Use DAB for color rendering. Hematoxylin again stained the nucleus and dehydrated the seal. Image-Pro Plus software was used to measure IOD values of NGF and IL-1β. Finally, collect data and plot with Graphpad prism.

### 2.8 Elisa assay

To evaluate the effects of *Camellia* extract on FN and IL-6 levels in mouse skin, homogenates were obtained from rat dorsal tissue. Follow the instructions and use the ELISA kit. 15 min after the end of the reaction, absorption at 450 wavelengths was measured.

### 2.9 Cell assay

To determine the appropriate concentration of *Camellia* extract for cell application by testing cell viability, MTT was used along with neutral red uptake. Cells capable of reducing MTT to insoluble formazan crystals are considered viable (Zhang et al., 2021). For this assay, RAW264.7 cells were inoculated into 96 well plates at a density of 1 × 10^5^ cells per well. 100 μL different tea extracts diluted with PBS (25 μmol/L) was added to the 96-well plate, and the blank group (culture medium) and the control group (cell + medium) were added with the same amount of medium. As a precaution against light incubation, each well was filled with 100 L of MTT working solution after 24 h of culture, supernatant was removed, 150 μL of dimethyl sulfoxide was added, and the 15-min period was dissolved by oscillation and optical density was determined by enzyme labeling at 490 nm.

Cell viability was also assessed by examining the accumulation of neutral red dye in viable cells. For logarithmic growth, RAW264.7 cell concentration was adjusted to 5 × 10^5^ per milliliter, incubation period of 24 h was carried out in each well of a 96-well plate, and supernatant was discarded thereafter, each well was added to the base medium with the highest cell activity selected by the MTT experiment. LPS group only added the medium 100 μL containing 30 μg/mL LPS, the blank group added the corresponding volume medium, incubated for 24 h, and abandoned the supernatant. After PBS washing, the final concentration of 0.1% neutral red solution 100 μL was added to each well to react 30 min, and the residual neutral red was washed. Using the equation below, the cell phagocytic index was calculated after lysis at 570 nm wavelength.

Phagocytic index = (total number of bacteria consumed by 100 phagocytic cells)/100.

### 2.10 Cell immune activity measured by neutral red assay

The concentration of RAW264.7 cells grown logically was adjusted to 5 × 10^5^ cells per milliliter, and 96-well plates were filled with 100 mL of bacterial solution each for 24 h, then the supernatant was discarded, and the basic medium of five components with the highest concentration of cell activity selected by MTT assay and LPS (30 μg/mL) were added to each hole (Guan et al., 2022). A medium containing 30 μg/mL LPS was added to the LPS group, and the blank medium was added to the blank group. Incubation for 24 h was followed by three washes with warm PBS after the supernatant was discarded, each well was filled with 100 mL of neutral red diluted to 0.1% and allowed to stand for 30 min. After that, the cells are pumped with a pipette gun and a warm PBS bath is given three times a night. A pipette gun was used to remove the supernatant, which was then washed three times with warm PBS (200 mL each time), and the remaining neutral red was washed away. The cell lysate was added with 100 μL/hole. Absorption measurement at 570 nm wavelength was performed on the split cell lysate after shaking for 15 min at room temperature.

### 2.11 Macrophage migration assay

Cell migration experiments were used to evaluate the effect of *Camellia* extract on cell migration and repair capacity. The experimental method was based on published literature and the experimental conditions were optimized (Yang et al., 2018). The back of the six hole plate should be marked with horizontal lines using a marker pen (compare it with a ruler), each hole passing through at least five lines, each line being uniform and parallel. Approximately 5–10 × 10^5^ cells were inoculated in the well. The next day, use the 20 μL gun head (sterilization) or toothpick, the black line screws on the back of the vertical orifice plate, so that the screws and the marking line intersect. After labeling, using sterile PBS, wash the cells 2–3 times, remove them, create a gap with your fingers, and then replace the fresh medium with serum-free or low-serum medium (<2%). The cells were cultured in an incubator at 37°C with 5% CO2. After 24 h, they were removed, viewed under a microscope, and photographed.

### 2.12 ROS release level assay

ROS level determination was used to evaluate *Camellia* extract’s effect on cellular redox activity, to understand its potential antioxidant effects. RAW264.7 cell suspensions were adjusted to 5 × 10^5^ cells per milliliter and added to wells of a six well plate in 2 mL each (Cheng et al., 2021). Supernatants were discarded after 24 h of culture and 1 mL of basic medium containing five tea extracts (40 μg/mL) was added to each well. After 2 h culture, LPS was added to make the final concentration of 1 μg/mL, and blank group and LPS model group were set. After the culture continued for 24 h, the supernatant was discarded and DCFH-DA (dissolved in PBS, the final concentration was 10 μM) was added. After 30 min, the cells were centrifuged from the six well plate. As a result, the fluorescent background was minimized with three PBS washes. After washing, the cells were re-suspended, 10 μL was placed in a counting plate, and then counted and photographed in an automatic fluorescent cell counter.

### 2.13 Immunofluorescence detection of NF-κB

As a nuclear transcription factor associated with inflammation, the levels of NF-κB after application of Camellia extract to cells can help understand its potential anti-inflammatory, antioxidant and other mechanisms of action. Adjusting the cell concentration to 1 × 10^5^ cells per milliliter, 1 mL of cell suspension was added to each well of the 24 well plate. After 24 h, the supernatant was discarded and 1 mL PBS was added to each well. Add 1 mL of different media-containing drug concentrations to each well and place in a 5% CO_2_ incubator for cell culture. Discard the supernatant after 24 h, add an appropriate amount of PBS and soak 3 times, each time for 5 min. Add 4% paraformaldehyde to fix for 15 min, then soak in PBS 3 times, each time for 5 min. Add 0.5% Triton X-100 for 10 min, then soak in PBS 3 times, each time for 5 min. Using 5% BSA (5 g BSA+100 mL TBST) to seal at room temperature for 1 h. Incubation of primary antibody: 200 μL of PPAR antibody (1:200) was added to each well of a 24 well plate, incubated overnight at 4°C, and then soaked with TBST for 3 times. Add the fluorescent secondary antibody (1:800) and incubate at room temperature in the dark for 1 h, then soak in TBST 3 times, each time for 5 min. DAPI staining solution (directly without dilution, 200 μL) was added and incubate for 10 min, then soak with PBS for 3 times. Place the 24-well plate in an inverted fluorescence microscope for photography.

### 2.14 Statistical analysis

Data are presented as mean ± standard error. Graphpad 8.0.2 statistical software (La Jolla, CA, United States) was used for all statistical analyses. For statistical comparison, the Tukey test and multivariate variance analysis (ANOVA) were used. *p < 0.05 and **p < 0.01 showed significant differences in temperature levels.

## 3 Result and discussion

### 3.1 Chemical compositions of five tea extracts species

Supplementary Material show the specific components of five kinds of *Camellias*. The ethanol extract was obtained by final analysis. They are caffeine, 1-stearyl glycerol, epigallocatechin gallate, D-quinic acid, 2-methylene-bis (4-methyl-6-tert-butylphenol), bis (4-ethyl benzyl) sorbitol, stearamide, (−)-Gallic catechin and 2-line 3-dihydroxypropyl 12-methyl triester. The main ingredients caffeine, D-quinic acid, mustard amide, 1-demethyleucine, gallocatechin gallate, choline, (−)-Gallic catechin and TOFA have anti-inflammatory activity. A high-performance liquid chromatography-mass spectrometry analysis was performed on different *Camellia* ethanol extracts.

### 3.2 Histological analysis of HE staining

**FIGURE 1:**
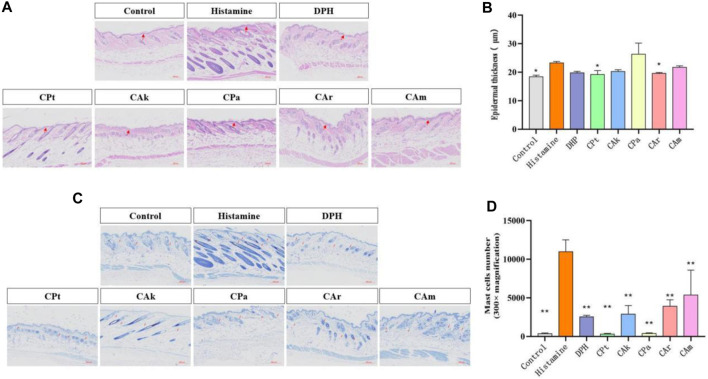
Histological evaluation of epidermal thickness of rat dorsal skin tissue. **(A)** Hematoxylin-eosin-stained rat dorsal epidermis. **(B)** Representative epidermal thickness for each treatment group. Statistical significance was assessed using one-way ANOVA followed by Duncan’s multiple-range test, revealing significant differences compared to the model group (**p < 0.01). Each value represents the mean ± SD of three mice. **(C)** Stained sections of mouse back skin tissue. **(D)** The number of mast cells in mouse skin tissue was quantified. Statistical analyses were performed using one-way ANOVA with Duncan’s multi-range test. Data are expressed as mean ± SD (*n* = 3 animals/group), with *p* < 0.05 and *p < 0.01 indicating statistical significance.

Epidermal hyperplasia is one of the characteristics of skin injuries caused by allergic reactions and is commonly used as an indicator to evaluate the inhibitory effect of drugs on epidermal hyperplasia. As shown in Figure 1A histological analysis of the tested skin showed that the epidermal hyperplasia of the positive group, CPt group, CAk group, CPa group, CAr group and CAm group were all lower than those of the model group, indicating that the local application of the five tea extracts tested could effectively inhibit epidermal hyperplasia caused by skin allergy. Skin thickness shown in Figure 1B shows that allergic reactions after histamine injection can lead to a significant increase in epidermal thickness compared to the blank group. Compared to the model group, treatment in the positive group, CPt, CAk, CPa, CAr, and CAm reduced skin epidermal thickness, but the effect of reducing skin epidermal thickness in the CPa group was slightly worse than in the other groups. The effect of the CAr group is similar to that of positive injection of diphenhydramine hydrochloride, which is well established to suppress epidermal hyperplasia due to skin allergies.

### 3.3 Toluidine blue for mast cells

The infiltration of mast cells is one of the hallmarks of skin inflammation due to allergic reactions. Therefore, the effect of CPt, CAk, CPa, CAr and CAm groups on mast cell penetration was analyzed by toluidine blue staining. As shown in Figure 1C There was a significant difference in mast cell counts in the dermis between the blank and model groups (**p < 0.01). Mast cells in the skin tissue of mice treated with CPt extract had the lowest degree of penetration compared to other components.

### 3.4 Inhibiting the production of inflammatory cytokines

**FIGURE 2:**
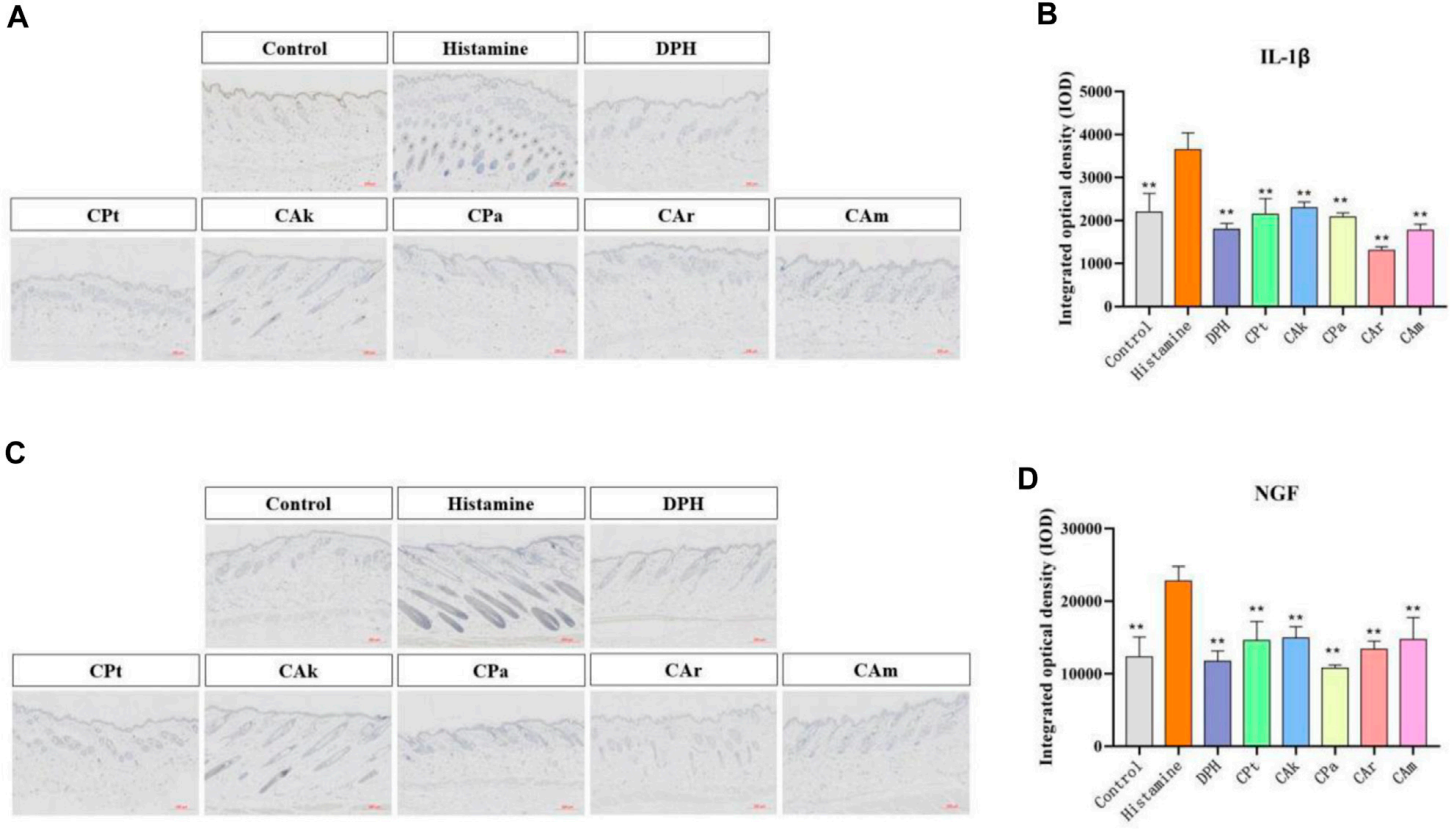
The expressions of IL-1β and nerve growth factor (NGF) in mouse skin were detected by immunohistochemical method. **(A)** Immunohistochemical staining of Interleukin-1β (IL-1β) treated with different compounds. **(B)** Immunohistochemical staining of nerve growth factor (NGF) treated with different compounds. **(C,D)** Quantification of relative optical density for IL-1β and NGF signals in dorsal skin samples from each assessment group was performed using image analysis software. Statistical significance was determined using one-way ANOVA followed by a post-hoc multi-range test, with **p < 0.01 indicating significant differences. Scale: 200:1. Each value represents the mean ± standard deviation of three mice.

The production of interleukin (IL-1β) and other inflammatory cytokines plays a crucial role in skin inflammation. Histamine-induced allergic itching significantly upregulates genes encoding various cytokines in the affected skin and significantly increases total serum. Evidence suggests it may increase the production of pro-inflammatory cytokines (including IL-4, IL-23, IL-1β and TNF-α). It can be seen from Figure 2A,B IL-1 expression was significantly increased in the model control group when compared to the study group (**p < 0.01). After treatment with five tea extracts, the expression of the model group was higher than that of the CPt, CAk, CPa, CAr, and CAm groups. The ability of CAr group to downregulate IL-1β expression was better than that of positive group.

The nerve growth factor (NGF) plays an important role in promoting the growth, differentiation, and maturation of peripheral and central neurons, maintaining normal nerve function after injury, and speeding up the healing process. The intensity of NGF expression in skin injury increases with the degree and grade of the injury. As shown in Figure 2C,D, the model group showed a significant increase in NGF expression compared to the blank group. When the model group was treated with CPa extract solution, NGF expression decreased significantly, with downregulated NGF expression in the CAk extract group approaching that in the positive group.

### 3.5 Immunofluorescence for NF-κB protein factor

**FIGURE 3:**
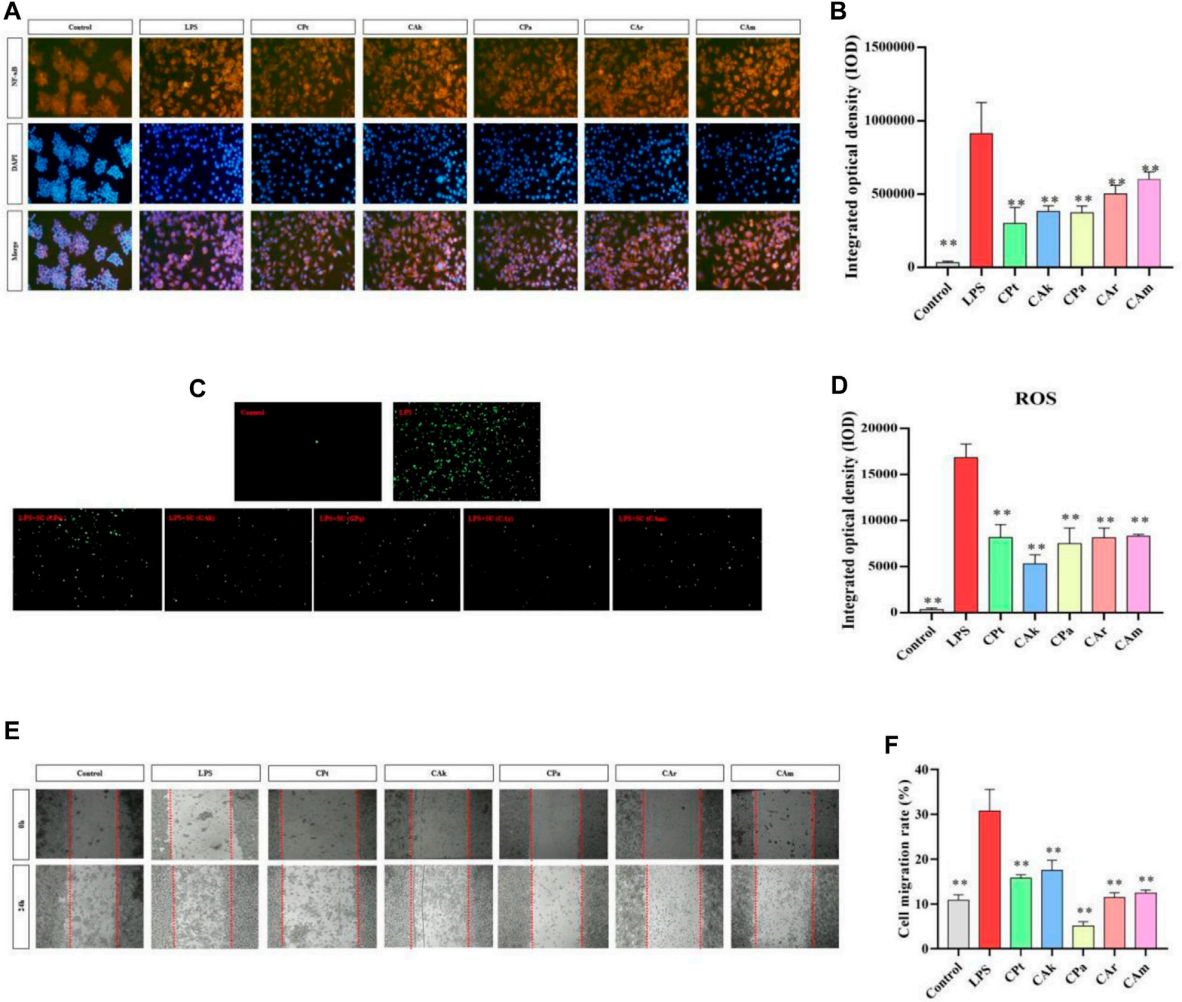
Effects of five Camellia tea extracts on LPS-induced activation of the NF-κB pathway, cellular oxidative stress and inhibition of macrophage migration. **(A,B)** Extracts affect RAW264.7 NF-B protein factor in macrophages, among them, orange section represented the level of NF-κB in cells, and blue section represented the cell level after DAPI staining. **(C,D)** Extracts affect RAW264.7 ROS in macrophages, and the green part represents ROS level in cells. **(E)** The images were taken at 0 and 24 h. **(F)** The migration cell numbers calculated at 24 h are displayed as bar graphs. Data represented mean ± SD (*n* = 3 per group). *p < 0.05 and **p < 0.01 compared to the LPS group. There were three groups with mean and SD. *p < 0.05 and **p < 0.01 when compared with control groups.

NF-κB regulates a series of pathological and physiological reactions, specifically, it is responsible for inflammatory and immune response, cell growth, differentiation, proliferation, and apoptosis. A signaling pathway called NF-*κ*B promotes the process of expression and release of inflammatory factors, including NGF and IL-6. When inhibited, NF-*κ*B can result in a reduction in inflammation. As shown in Figure 3A,B, when cells were stimulated by LPS, the secretion of inflammatory cytokine protein increased, and there was a significant upregulation in the expression of NF-*κ*B protein factor compared to the Control group. After co-culture with different extracts, the integrated optical density (IOD) of cells treated with five kinds of tea extracts decreased significantly. It is suggested that five kinds of tea extracts have significant effects on reducing cell inflammation and the content of NF-κB protein factor, and the experimental results of five kinds of tea extracts are statistically significant.

### 3.6 Decreasing cellular oxidative stress

ROS production through oxygen metabolism is a natural process and plays an important role in signaling and homeostasis within the cell (Lion et al., 2021). However, intracellular ROS levels are dramatically increased under LPS stimulation. This can lead to severe damage to cell structure through oxidative stress, and reducing ROS expression reduces the body’s inflammatory response. As shown in Figure 3C,D, under the influence of LPS, there was a significant increase in ROS release in macrophages compared to blank cells (**p < 0.01), indicating that ROS release in macrophages would be significantly increased after the LPS-induced cell inflammation model. Compared to lipopolysaccharide, different extracts significantly reduced ROS released by macrophages in co-culture cells. (Model group), indicating that the five tea extract groups in this study could significantly reduce the inflammatory response and intracellular ROS release induced by lipopolysaccharide, and better resist lipopolysaccharide-induced inflammatory stimulation. The experimental data are statistically significant for five tea extracts.

### 3.7 Decreasing the migration of LPS stimulated macrophages

As shown in Figure 3E,F, after administration of LPS, there was a significant difference in cell migration between the model group and the blank group, indicating that endotoxin caused cell inflammation in the model group. Five types of tea extracts were found to have a significant decrease in cell migration compared to the model group, with CPa group having the most significant ability to reduce cell migration. This shows that these five tea extracts have anti-inflammatory effects.

### 3.8 Immunological activities of five tea extracts

**FIGURE 4:**
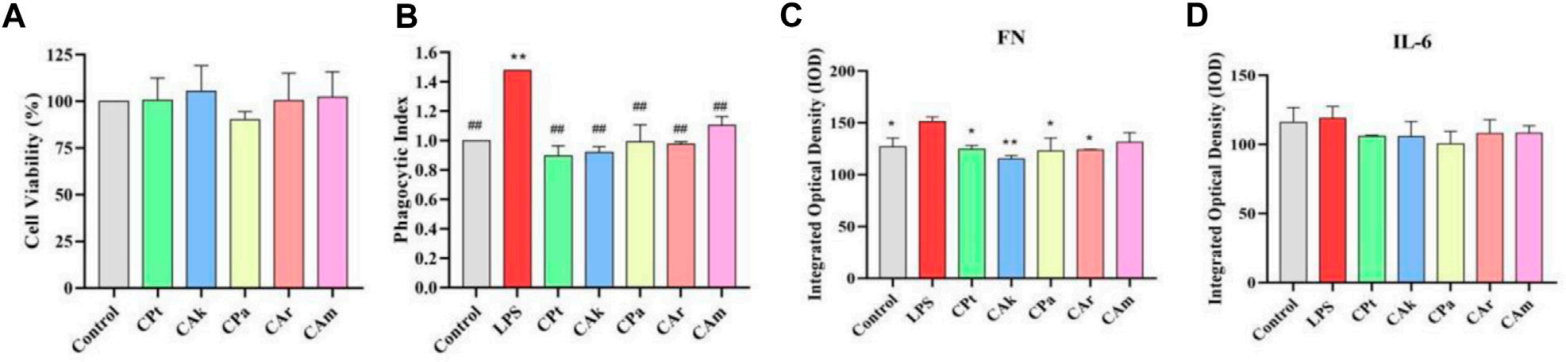
Immune activity and inhibitory effect of five tea extracts on cellular inflammatory factors. **(A)** Cell viability was determined by MTT assay. **(B)** A neutral red phagocytosis index was measured to assess cellular phagocytic activity. **(C,D)** The inhibition levels of fibronectin (FN) protein and the cytokine Interleukin-6 (IL-6) were quantified by ELISA. Statistical analyses for all parameters were performed using one-way ANOVA followed by a suitable post-hoc test to identify significant differences between groups. Data are presented as mean ± SD (*n* = 3 per group), with *p < 0.05 and **p < 0.01 indicating statistical significance when compared with the LPS-treated control group.

Phagocytosis refers to the ability of macrophages to enhance the body’s ability to fight infection by phagocytosis of invading pathogens and aging and deformed cells in the body, and is a critical measure of macrophage activity. Macrophages exert immune effects by phagocytosis to remove foreign bodies, pathogens, and foreign cells. As shown in Figure 4A, MTT assay showed that extracts had no cytotoxicity to RAW 264.7 cells in different groups. As shown in Figure 4B, 24 h after LPS stimulation alone on RAW264.7 macrophages, there was a significant increase in phagocytic index in the LPS group compared to the blank group, indicating that macrophages can phagocytose more easily. After the cells were treated with five tea extracts for 24 h, it was found that phagocytosis capacity of phagocytes could be downregulated in each dose group.

### 3.9 Inhibiting cellular Inflammatory cytokines

Implicated protein (FN) is a widely existing protein active substance in the human body, which can mobilize the immune cell system to remove harmful substances in damaged tissues. It has the same effect as growth factors, but the effect is mild without side effects. Increased expression of the implicated protein (FN) is beneficial to improving the body’s ability to resist inflammation. As shown in Figure 4C, the concentration of FN in macrophages was significantly increased after LPS stimulation compared to the blank population. After addition of extracts, a significant decrease in FN was observed in each group compared to the inflammatory model containing LPS, suggesting that the five tea extracts could reduce FN.

Inflammatory factors such as IL-6 are produced by mononuclear macrophages. Reducing infection or immunity increases IL-6 levels, which in turn upregulates the expression of inducible nitric oxide synthase, which in severe cases can cause tissue or organ damage. Inhibition of IL-6 expression reduces the level of inflammation in the body. The concentration of IL-6 in macrophages stimulated by LPS was significantly increased compared to the blank population. As shown in Figure 4D, compared to the blank group, LPS stimulation resulted in a significant increase in IL-6 levels in macrophages, while levels of IL-6, a cytokine associated with inflammation, decreased, suggesting that extracts used may reduce IL-6 levels in macrophages in skin tissue samples.

## 4 Discussion

Tea is widely used as a natural herbal drink that plays an anti-allergy role not only in allergy prevention, but also in allergy treatment. These also include Camellia plants; however, different teas have different effects, and evidence from existing studies evaluating tea’s efficacy in treating histamine-induced atopic dermatitis is still limited to determine the mature mechanism (Li et al., 2021).

At present, the effective anti-inflammatory ingredients and specific content of different types of tea have not been widely reported. To explore the effective ingredients in tea extracts for allergy treatment, GC-MS was used to identify ethanol extracts from five teas. Major ingredients with proven anti-inflammatory activity were identified: caffeine, D-quinic acid, mustard amide, 1-demethyleucine, gallocatechin gallate, choline, (−)-Gallic catechin, and TOFA.

Skin allergy can lead to skin hyperplasia due to local skin vascular dilation or the release of inflammatory substances (Viljanen et al., 2005). HE staining showed that the skin thickening of the mice epidermis was reduced after treatment with tea extract. In a mouse model of histamine-induced atopic dermatitis, skin allergic reactions were mitigated by reducing proliferative infiltration of mast cells and reducing degranulation of mast cells (Song et al., 2014), as confirmed by toluidine blue staining of five tea extracts. As skin damage causes the release of cytokines (such as TNF-α and IL-1β), these cytokines activate cells such as keratinocytes and fibroblasts, which release NGF (Piazza et al., 2022). Immunohistochemical staining was used to measure the expression levels of IL-1β and NGF, which showed that five tea extracts could downregulate the inflammatory effect of cells by down-regulating the expression levels of IL-1β and NGF. In conclusion, five tea extracts can resist histamine-induced allergic dermatitis symptoms by inhibiting inflammatory factors, inhibiting mast cell penetration, and delamination. Among them, five tea extracts have advantages in the repair of atopic dermatitis and have the potential of anti-allergic substances.

The transcription factor NF-κB plays a crucial role in regulating various aspects of innate and adaptive immune functions, serving as a pivotal mediator of inflammatory responses. S signaling is essential for the initiation and propagation of inflammatory pathways, regulating the expression of multiple inflammatory genes through its nuclear localization (Nguyen et al., 2022). The results of immunofluorescence assay confirmed the inhibitory effect of five tea extracts on NF-κB signaling pathway in mouse RAW264.7 macrophage inflammation model induced by LPS. MTT assay and screw migration were used to determine that tea extracts in the effective concentration range were not only noncytotoxic, but also downregulated cytophagocytosis. IL-6 is an important cytokine that regulates the body’s immunity and can induce CRP formation and inflammatory cell aggregation (Del Giudice and Gangestad, 2018). As a result of its increase, inflammatory mediators are released in patients with atopic dermatitis, which worsen inflammatory symptoms. ELISA confirmed the decreased effect of tea extract on IL-6 and FN expression to reduce the protective effect of mouse macrophages against LPS-induced inflammation. In addition, we detected reduced ROS levels in mouse macrophages following treatment with the five tea extracts, suggesting a downregulation of ROS expression and a consequent reduction in cellular inflammatory responses (Lion et al., 2021). These results collectively indicate a potential improvement in the immunosuppressive effects of the five tea extracts on mouse macrophages, providing protection against lipopolysaccharide-induced cellular inflammation.

Our study demonstrated the efficacy of tea extracts from five Camellia species in ameliorating histamine-induced atopic dermatitis, reducing inflammation levels in mouse skin, and mitigating oxidative stress and inflammation in macrophages. While this investigation provides preliminary evidence for the therapeutic potential of these tea extracts in an animal model of histamine-induced atopic dermatitis and in a mouse macrophage RAW264.7 cell inflammation model induced by LPS, further exploration is warranted to elucidate the underlying molecular mechanisms responsible for the protective effects of Camellia tea extracts on the skin. In conclusion, our findings hold promise for advancing research in related fields and may offer novel therapeutic strategies for managing histamine-induced allergic dermatitis.

## Data Availability

The original contributions presented in the study are included in the article/Supplementary Material, further inquiries can be directed to the corresponding authors.
