# Supplementary material for: Tea for histamine anti-allergy: component analysis of tea extracts and potential mechanism for treating histamine anti-allergy
Source: Front Pharmacol. 2024 May 30;15:1296190. doi: 10.3389/fphar.2024.1296190 (PMC11169817; doi:10.3389/fphar.2024.1296190)
Supplement: Supplementary file 1 [file Table1.docx]

Supplementary Table.1 Table of chemical constituents of five *Camellia* ethanol extracts

| NO | Identity | RT [min] | Adducta | Main fragment ions (relative intensity) | Measured mass(m/z) | Formula |
| --- | --- | --- | --- | --- | --- | --- |
| 1 | (-)-Gallocatechin | 7.157 | [M+H]+ | 139.03857,163.03845 | 306.07345 | C_15_H_14_O_7_ |
| 2 | (±)9(10)-DiHOME | 21.351 | [M-H]- | 171.10243,277.21719,295.22739 | 314.24565 | C_18_H_34_O_4_ |
| 3 | (±)9-HpODE | 21.124 | [M-H]- | 171.10257,211.13394,293.21237 | 312.23027 | C_18_H_32_O_4_ |
| 4 | (2R,3R,4S,5S,6R)-2-[(3Z)-hex-3-en-1-yloxy]-6-(hydroxymethyl)oxane-3,4,5-triol | 13.612 | [M+H]+ | 88.07587 | 262.14092 | C_12_H_22_O_6_ |
| 5 | (2R,3R,4S,5S,6R)-2-{[(2E,6R)-6-hydroxy-2,6-dimethylocta-2,7-dien-1-yl]oxy}-6-(hydroxymethyl)oxane-3,4,5-triol | 15.749 | [M+H]+ | 203.05202 | 332.18263 | C_16_H_28_O_7_ |
| 6 | (3beta,9xi)-3-(beta-D-Glucopyranosyloxy)-14-hydroxycard-20(22)-enolide | 22.72 | [M+H]+ | 520.62354 | 536.2954 | C_29_H_44_O_9_ |
| 7 | (3R,4R)-3-({4-[(3,5-Dimethyl-1,2-oxazol-4-yl)methoxy]benzoyl}amino)-4-hydroxy-N-[4-(trifluoromethoxy)phenyl]-1-azepanecarboxamide | 13.752 | [M+H]+ | 203.05209,204.07527 | 562.20167 | C_27_H_29_F_3_N_4_O_6_ |
| 8 | (4-chlorophenyl)[4,6-dimethyl-3-(1H-pyrrol-1-yl)thieno[2,3-b]pyridin-2-yl]methanone | 1.558 | [M+H]+ | 349.05188 | 366.05542 | C_20_H_15_ClN_2_OS |
| 9 | [3,4,5-trihydroxy-6-(3,4,5-trihydroxybenzoyl)oxyoxan-2-yl]methyl 3,4,5-trihydroxybenzoate | 9.571 | [M-H]- | 125.0242,169.01402,211.02478,271.0459 | 484.08527 | C_20_H_20_O_14_ |
| 10 | 1-(3,4-dichlorophenoxy)-3-({2-[(5-propylpyrimidin-2-yl)amino]ethyl}amino)propan-2-ol | 13.947 | [M+H]+ | 202.05974 | 398.13347 | C_18_H_24_Cl_2_N_4_O_2_ |
| 11 | {(1R,2R)-2-[(2Z)-5-(Hexopyranosyloxy)-2-penten-1-yl]-3-oxocyclopentyl}acetic acid | 13.258 | [M-H]- | 59.01374,71.01371,89.02423,387.16608 | 388.17366 | C_18_H_28_O_9_ |
| 12 | 1-(3,4-dichlorophenoxy)-3-({2-[(5-propylpyrimidin-2-yl)amino]ethyl}amino)propan-2-ol | 13.947 | [M+H]+ | 202.05974 | 398.13347 | C_18_H_24_Cl_2_N_4_O_2_ |
| 13 | 1,2-Dipalmitoylphosphatidylglycerol | 24.197 | [M+H]+ | 745.50549 | 722.51659 | C_38_H_75_O_10_P |
| 14 | 1-Stearoylglycerol | 24.426 | [M+H]+ | 57.07054,71.08604,95.08577,109.10127 | 358.30741 | C_21_H_42_O_4_ |
| 15 | 2,2'-Methylenebis(4-methyl-6-tert-butylphenol) | 23.422 | [M-H]- | 163.1127 | 340.24028 | C_23_H_32_O_2_ |
| 16 | 2,3-Dihydroxybenzoic acid | 2.866 | [M-H]- | 109.02929,153.01913 | 154.02644 | C_7_H_6_O_4_ |
| 17 | 2,3-dihydroxypropyl 12-methyltridecanoate | 23.053 | [M+H]+ | 57.07052,71.08601,95.08573,285.24155 | 302.24482 | C_17_H_34_O_4_ |
| 18 | 4-(4-hydroxy-2,6,6-trimethyl-3-{[(2R,3R,4S,5S,6R)-3,4,5-trihydroxy-6-(hydroxymethyl)oxan-2-yl]oxy}cyclohex-1-en-1-yl)butan-2-one | 13.853 | [M+H]+ | 107.08560,149.09576,191.14252 | 388.20876 | C_19_H_32_O_8_ |
| 19 | 4-Hydroxybenzaldehyde | 14.74 | [M+H]+ | 123.04399 | 122.0368 | C_7_H_6_O_2_ |
| 20 | 4-tert-Amylphenol | 23.423 | [M-H]- | 91.77166,101.23451,133.63811,151.86169 | 164.11852 | C_11_H_16_O |
| 21 | 5-(Ethylsulfonyl)-2-[(3S)-1-(4-methoxybenzyl)-3-pyrrolidinyl]-1,3-benzoxazole | 14.273 | [M+H]+ | 204.07503 | 400.14894 | C_21_H_24_N_2_O_4_S |
| 22 | 5-Hydroxymethyl-2-furaldehyde | 0.981 | [M+H]+ | 81.03387,109.02845,113.01891,127.03877 | 126.03163 | C_6_H_6_O_3_ |
| 23 | 5-OxoETE | 22.342 | [M+H]+ | 301.21295 | 318.21647 | C_20_H_30_O_3_ |
| 24 | 6,8-dihydroxy-3-(10-hydroxyundecyl)-3,4-dihydro-1H-2-benzopyran-1-one | 18.825 | [M+H]+ | 351.17682 | 350.20604 | C_20_H_30_O_5_ |
| 25 | 6-hydroxy-3,5a,9-trimethyl-2H,3H,3aH,4H,5H,5aH,6H,7H,9aH,9bH-naphtho[1,2-b]furan-2-one | 19.232 | [M+H]+ | 251.16115 | 250.15386 | C_15_H_22_O_3_ |
| 26 | 9-Oxo-10(E),12(E)-octadecadienoic acid | 22.043 | [M+H]+ | 67.05479,93.0703,179.14267,277.21555 | 294.21877 | C_18_H_30_O_3_ |
| 27 | Adenosine | 1.259 | [M+H]+ | 136.06148 | 267.09582 | C_10_H_13_N_5_O_4_ |
| 28 | Caffeic acid | 16.45 | [M-H]- | 71.04964,85.02876,287.05426 | 432.1051 | C_21_H_20_O_10_ |
| 29 | AL 8810 Methyl ester | 17.217 | [M+H]+ | 439.22925 | 416.24012 | C_25_H_33_FO_4_ |
| 30 | Astragalin | 15.648 | [M+H]+ | 85.02872,287.05411 | 448.1001 | C_21_H_20_O_11_ |
| 31 | Bicyclo Prostaglandin E2 | 20.872 | [M+H]+ | 335.21844 | 334.21122 | C_20_H_30_O_4_ |
| 32 | Bis(4-ethylbenzylidene)sorbitol | 20.102 | [M+H]+ | 119.08537 | 414.20329 | C_24_H_30_O_6_ |
| 33 | Corchorifatty acid F | 12.44 | [M-H]- | 90.92448,121.02946,135.04504 | 180.04215 | C_9_H_8_O_4_ |
| 34 | Caffeine | 9.965 | [M+H]+ | 110.07123,138.06578 | 194.07998 | C_8_H_10_N_4_O_2_ |
| 35 | Choline | 0.933 | [M+H]+ | 60.08139,87.04436 | 103.09989 | C_5_H_13_NO |
| 36 | Citric acid | 1.078 | [M-H]- | 85.02936,87.00860,111.00860,191.05597 | 192.02703 | C_6_H_8_O_7_ |
| 37 | Gallic acid | 18.828 | [M-H]- | 85.02938,171.10254,211.1339,229.14445 | 328.22506 | C_18_H_32_O_5_ |
| 39 | D-(-)-Quinic acid | 0.982 | [M-H]- | 85.02937,111.00861 | 192.06323 | C_7_H_12_O_6_ |
| 40 | D-(+)-Pyroglutamic Acid | 1.052 | [M+H]+ | 84.04473,84.08109,130.04965,130.08603 | 129.04247 | C_5_H_7_NO_3_ |
| 41 | Docosanamide | 25.679 | [M+H]+ | 57.07051,72.04485 | 339.34927 | C_22_H_45_NO |
| 42 | Elaidic acid | 24.399 | [M-H]- | 273.0181 | 282.25582 | C_18_H_34_O_2_ |
| 43 | Epigallocatechin gallate | 12.712 | [M+H]+ | 139.0387,151.03868,153.0179 | 458.08481 | C_22_H_18_O_11_ |
| 44 | EQH | 13.041 | [M+H]+ | 413.17728 | 412.17001 | C_16_H_24_N_6_O_7_ |
| 45 | Erucamide | 25.13 | [M+H]+ | 69.07039,83.08589,97.10138 | 337.33354 | C_22_H_43_NO |
| 46 | Esculetin | 11.179 | [M+H]+ | 123.04393,135.04378 | 178.02626 | C_9_H_6_O_4_ |
| 47 | Fisetin | 16.457 | [M+H]+ | 287.05405,287.12436 | 286.04688 | C_15_H_10_O_6_ |
| 48 | Gallic acid | 14.771 | [M-H]- | 125.02422,169.01392 | 170.02037 | C_7_H_6_O_5_ |
| 49 | Glabridin | 20.506 | [M+H]+ | 123.04388,149.05931,189.09041 | 324.1356 | C_20_H_20_O_4_ |
| 50 |  | 14.771 |  | 125.02422,169.01392 | 170.02037 |  |
| 51 | Gluconic acid | 0.972 | [M-H]- | 59.01374,75.00859,87.00861,129.01921 | 196.05806 | C_6_H_12_O_7_ |
| 52 | Grosvenorine | 15.585 | [M+H]+ | 71.04963,85.02872,287.05414 | 740.21585 | C_33_H_40_O_19_ |
| 53 | Heroin-d3 | 12.388 | [M+H]+ | 367.14224 | 372.17511 | C_21_H_20_[_2_]H_3_NO_5_ |
| 54 | Isophorone | 16.232 | [M+H]+ | 69.03402,139.11147 | 138.10425 | C_9_H_14_O |
| 55 | Isoquercitrin | 14.884 | [M+H]+ | 85.02869,303.04889 | 464.09494 | C_21_H_20_O_12_ |
| 56 | Isovitexin | 14.683 | [M+H]+ | 283.0592,313.06964,337.06946,367.08014 | 432.10491 | C_21_H_20_O_10_ |
| 57 | Kaempferol | 15.651 | [M+H]+ | 153.01782,287.05408 | 286.04693 | C_15_H_10_O_6_ |
| 58 | Kaempferol-3-O-rutinoside | 15.657 | [M+H]+ | 71.04961,85.02871,287.05405 | 594.15805 | C_27_H_30_O_15_ |
| 59 | L-Glutamic acid | 23.916 | [M+H]+ | 84.04474,102.0551,130.04968 | 280.24019 | C_5_H_9_NO_4_ |
| 60 | Linoleic acid | 23.244 | [M-H]- | 179.65894 | 323.28161 | C_18_H_32_O_2_ |
| 61 | Linoleoyl Ethanolamide | 1.394 | [M+H]+ | 62.0606,67.05473,81.07021,95.08569 | 131.09454 | C_20_H_37_NO_2_ |
| 62 | L-Norleucine | 2.244 | [M+H]+ | 69.07042,72.04491,86.09676,114.09135 | 165.07867 | C_6_H_13_NO_2_ |
| 63 | L-Phenylalanine | 17.781 | [M+H]+ | 103.05441,120.08076 | 286.04742 | C_9_H_11_NO_2_ |
| 64 | Luteolin | 0.971 | [M-H]- | 285.04047 | 117.07899 | C_15_H_10_O_6_ |
| 65 | L-Valine | 0.978 | [M+H]+ | 58.06574,59.07355,72.08122,118.08614 | 95.98792 | C_5_H_11_NO_2_ |
| 66 | Methanesulfonic acid | 19.407 | [M-H]- | 79.9572,94.98067 | 352.22175 | CH_4_O_3_S |
| 67 | Methoxyacetyl fentanyl | 0.922 | [M+H]+ | 343.22748 | 147.05287 | C_22_H_28_N_2_O_2_ |
| 68 | Methyl (2R,4S,6S,12bR)-4-(4-fluorophenyl)-2-{[2-(4-morpholinyl)ethyl]amino}-1,2,3,4,6,7,12,12b-octahydroindolo[2,3-a]quinolizine-6-carboxylate | 23.019 | [M+H]+ | 507.27094 | 506.26366 | C_29_H_35_FN_4_O_3_ |
| 69 | Monolaurin | 22.113 | [M+H]+ | 297.20282 | 274.21374 | C_15_H_30_O_4_ |
| 70 | Monoolein | 24.001 | [M+H]+ | 69.0704,81.07028,265.25189,339.2886 | 356.29175 | C_21_H_40_O_4_ |
| 71 | Morin | 14.636 | [M+H]+ | 153.01784,229.04883 | 302.04181 | C_15_H_10_O_7_ |
| 72 | Myricetin | 13.899 | [M+H]+ | 153.01787,217.04898,245.04375,273.03839 | 318.03673 | C_15_H_10_O_8_ |
| 73 | myricetin 3-O-beta-D-galactopyranoside | 13.902 | [M+H]+ | 85.02874,153.01787,319.04391 | 480.08991 | C_21_H_20_O_13_ |
| 74 | N-Acetyl-DL-tryptophan | 12.849 | [M-H]- | 74.02461,116.03517,116.0504,203.08252 | 246.10033 | C_13_H_14_N_2_O_3_ |
| 75 | Neochlorogenic acid | 9.668 | [M-H]- | 135.045,173.0453,179.81998,191.05597 | 354.09483 | C_16_H_18_O_9_ |
| 76 | Nicotinamide | 1.08 | [M+H]+ | 80.04984,123.05513 | 122.04798 | C_6_H_6_N_2_O |
| 77 | NP-000587 | 6.934 | [M-H]- | 119.05,163.03986,191.05592 | 338.1 | C_16_H_18_O_8_ |
| 78 | NP-002113 | 20.754 | [M+H]+ | 314.97702 | 332.1956 | C_20_H_28_O_4_ |
| 79 | NP-008993 | 20.804 | [M-H]- | 311.22372 | 314.24587 | C_18_H_34_O_4_ |
| 80 | NP-010776 | 14.889 | [M+H]+ | 137.02303,153.01785,229.04884 | 302.04185 | C_15_H_10_O_7_ |
| 81 | NP-020139 | 2.433 | [M-H]- | 108.02152,152.01129 | 316.07956 | C_13_H_16_O_9_ |
| 82 | NP-020760 | 17.579 | [M+H]+ | 203.05202 | 418.25592 | C_21_H_38_O_8_ |
| 83 | NP-021781 | 21.264 | [M+H]+ | 367.2103 | 344.25547 | C_19_H_36_O_5_ |
| 84 | NP-021797 | 18.964 | [M+H]+ | 237.14546 | 214.1564 | C_12_H_22_O_3_ |
| 85 | Nα-({(3R,4R,5R)-4,5-dihydroxy-3-[(3-pyridinylcarbonyl)amino]-1-cyclohexen-1-yl}carbonyl)-L-tyrosinamide | 11.664 | [M-H]- | 59.01376,89.02432,207.10255,387.16629 | 440.16493 | C_22_H_24_N_4_O_6_ |
| 86 | Oleanolic acid | 23.634 | [M-H]- | 455.31732 | 456.35933 | C_30_H_48_O_3_ |
| 87 | Oleoyl ethylamide | 24.385 | [M+H]+ | 72.04486,114.09129 | 309.30232 | C_20_H_39_NO |
| 88 | Palmitic acid | 24.426 | [M-H]- | 237.09412 | 256.24022 | C_16_H_32_O_2_ |
| 89 | PEG n11 | 14.425 | [M+H]+ | 89.05999,133.08569,177.11162,503.30521 | 502.29807 | C_22_H_46_O_12_ |
| 90 | PEG n12 | 14.765 | [M+H]+ | 89.06007,133.08583,177.11179 | 546.32419 | C_24_H_50_O_13_ |
| 91 | PEG n8 | 12.834 | [M+H]+ | 89.06001,133.08571,177.11154 | 370.21938 | C_16_H_34_O_9_ |
| 92 | Phenylethyl 2-Glucoside | 12.702 | [M+H]+ | 85.02872,127.0388,145.04916,163.05959 | 284.12516 | C_14_H_20_O_6_ |
| 93 | Pipecolic acid | 0.983 | [M+H]+ | 70.06561,84.08109,130.08601 | 129.07886 | C_6_H_11_NO_2_ |
| 94 | Pyrogallol | 1.499 | [M-H]- | 69.03442,97.02933 | 126.03152 | C_6_H_6_O_3_ |
| 95 | Quercetin | 17.04 | [M-H]- | 107.01374,121.0293,151.00346,301.03543 | 302.04213 | C_15_H_10_O_7_ |
| 96 | Rutin | 14.888 | [M+H]+ | 71.04961,85.0287,303.04892 | 610.15276 | C_27_H_30_O_16_ |
| 97 | Salicylic acid | 13.534 | [M-H]- | 93.03438,137.02417 | 138.03149 | C_7_H_6_O_3_ |
| 98 | Stearamide | 24.171 | [M+H]+ | 284.29419 | 283.28694 | C_18_H_37_NO |
| 99 | Stearic acid | 24.86 | [M-H]- | 223.02457 | 284.27155 | C_18_H_36_O_2_ |
| 100 | Succinic acid | 1.242 | [M-H]- | 73.02934,117.05539 | 118.02647 | C_4_H_6_O_4_ |
| 101 | Theobromine | 3.403 | [M+H]+ | 181.07155 | 180.06439 | C_7_H_8_N_4_O_2_ |
| 102 | Tiliroside | 17.166 | [M+H]+ | 91.0545,119.04909,147.04372,287.05414 | 594.13684 | C_30_H_26_O_13_ |
| 103 | TOFA | 23.056 | [M+H]+ | 325.2341 | 324.22701 | C_19_H_32_O_4_ |
| 104 | Tridemorph | 24.453 | [M+H]+ | 57.07051,58.02936,88.07596,102.09145 | 297.3024 | C_19_H_39_NO |
| 105 | Vicenin II | 12.962 | [M+H]+ | 295.05914,325.06952,337.0694,457.112 | 594.15772 | C_27_H_30_O_15_ |
| 106 | Vicenin III | 13.711 | [M+H]+ | 295.0592,325.06964,379.07999,427.10147 | 564.14715 | C_26_H_28_O_14_ |
| 107 | Vitexin | 14.221 | [M+H]+ | 283.05927,284.06696,313.06967,415.10123 | 432.10503 | C_21_H_20_O_10_ |
| 108 | Vitexin rhamnoside | 14.36 | [M+H]+ | 283.05914,313.06958,433.11166 | 578.16279 | C_27_H_30_O_14_ |
| 109 | α,α-Trehalose | 0.942 | [M-H]- | 59.01375,71.01369,89.02427,179.05592 | 342.11622 | C_12_H_22_O_11_ |
| 110 | δ-Valerolactam | 2.49 | [M+H]+ | 56.05013,82.06552,100.07584 | 99.06868 | C_5_H_9_NO |
